# Supplementary material for: Dietary Supplementation of Aspirin Promotes Drosophila Defense against Viral Infection
Source: Molecules. 2023 Jul 9;28(14):5300. doi: 10.3390/molecules28145300 (PMC10385701; doi:10.3390/molecules28145300)
Supplement: Supplementary file 1 [file molecules-28-05300-s001.zip › molecules-2406326-supplementary.pdf]

Figure S1

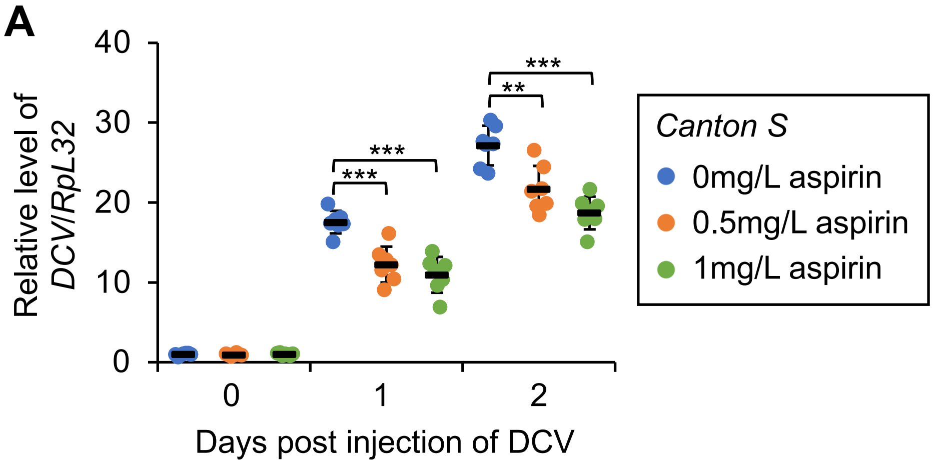

Figure S1. Dietary supplementation of aspirin limits DCV load in *Canton S* flies.

(A) Male *Canton S* adults were raised on standard fly medium supplemented with or without aspirin as indicated. Flies were infected with DCV, followed by RT-qPCR assays to monitor the viral load at indicated time points. Data are shown as mean plus standard errors. Each dot represents one independent replicate. **, $p<0.01$; ***, $p<0.001$.
